# TGF-*β*3 Induces Autophagic Activity by Increasing ROS Generation in a NOX4-Dependent Pathway

**DOI:** 10.1155/2019/3153240

**Published:** 2019-12-31

**Authors:** Yun Zhang, Hong-Mei Tang, Chun-Feng Liu, Xie-Fang Yuan, Xiao-Yun Wang, Ning Ma, Guo-Feng Xu, Song-Ping Wang, Jun Deng, Xing Wang

**Affiliations:** ^1^Inflammation & Allergic Diseases Research Unit, Affiliated Hospital of Southwest Medical University, Luzhou, 646000 Sichuan, China; ^2^First Department of Respiratory Disease, Affiliated Hospital of Southwest Medical University, Luzhou, 646000 Sichuan, China

## Abstract

Higher concentrations of reactive oxygen species (ROS) have been associated with epithelial cell damage, cell shedding, and airway hyperresponsiveness. Previous studies have indicated that transforming growth factor-beta (TGF-*β*) mediates ROS production and NADPH oxidase (NOX) activity. In our previous study, we also observed that TGF-*β*3 increases mucus secretion in airway epithelial cells in an autophagy-dependent fashion. Although it is well known that the relationship between ROS and autophagy is cell context-dependent, the exact mechanism of action remains unclear. The following study examined whether ROS act as upstream of autophagy activation in response to TGF-*β*3 induction. Using an allergic inflammation mouse model induced by house dust mite (HDM), we observed elevated lung amounts of TGF-*β*3 accompanied by increased ROS levels. And we found that ROS levels were elevated and NOX4 expression was increased in TGF-*β*3-induced epithelial cells, while the lack of NOX4 in the epithelial cells could reduce ROS generation and autophagy-dependent MUC5AC expression treated with TGF-*β*3. Furthermore, our studies demonstrated that the Smad2/3 pathway was involved in TGF-*β*3-induced ROS generation by promoting NOX4 expression. The inhibition of ROS generation by N-Acetyl-L-cysteine (NAC) resulted in a decrease in mucus expression and autophagy activity *in vivo* as well as *in vitro*. Finally, TGF-*β*3-neutralizing antibody significantly reduced the ROS generation, mucus expression, and autophagy activity and also decreased the phosphorylation of Smad2 and Smad3. Taken together, the obtained results revealed that persistent TGF-*β*3 activation increased ROS levels in a NOX4-dependent pathway and subsequently induced autophagy as well as MUC5AC expression in the epithelial cells.

## 1. Introduction

Reactive oxygen species (ROS), identified as signaling molecules that regulate both cell survival and cell death, have an important role in the pathogenesis of airway inflammation and tissue injury associated with asthma [1–3]. At higher concentration, ROS can influence airway cells and reproduce many of the pathophysiologic features, including epithelial cell damage, cell shedding, and airway hyperresponsiveness [1–3]. Autophagy is a dynamic process responsible for the turnover of organelles and long-lived proteins, which plays a crucial role in maintaining cellular integrity and adaptation to adverse environments [4]. In mammals, the net amount of microtubule-associated protein 1 light chain 3*β* II (LC3B-II) is a critical hallmark for monitoring autophagy [5]. A current paradigm positions ROS upstream of autophagy activation in response to cell stress [6–9]. In contrast, autophagy has an effect on the intracellular ROS generation [10–12]. House dust mite (HDM) is a major perennial allergen source and a significant cause of allergic rhinitis and allergic asthma [13]. HDM can directly lead to the production of reactive oxygen [14]. Studies have suggested that the relationship between ROS and autophagy is cell context-dependent and that ROS production regulates MUC5AC expression [15]; MUC5AC is considered a marker of mucus cell hyperplasia or metaplasia because of its high expression in mucus-secreting goblet cells. In addition, the environmental stimulus and cytokines have shown to induce autophagy activation and subsequently the mucus hypersecretion, including cigarette smoke extracts (CSE), fine particulate matter (PM2.5), and IL-13 [16–18]. Moreover, CSE regulate airway mucus hypersecretion via ROS-dependent autophagy activation [16]. Furthermore, TGF-*β*1/2 has shown the ability to simultaneously induce autophagy and ROS levels [19–22]. TGF-*β*1 promotes autophagy through the generation of ROS in renal tubular epithelial cells [23]. Moreover, previous observations have suggested that autophagy is considered as an important aspect of biological effects of TGF-*β*3 in modulating airway mucus hypersecretion [24]. However, the exact mechanism of the relationship between TGF-*β*3-induced autophagy and ROS remains unclear. Thus, these studies provide a deserving consideration whether TGF-*β*3 has the effect on the production of ROS.

Mitochondria and the NOX family of nicotinamide adenine dinucleotide phosphate (NADPH) oxidases are the two major sources of ROS that are induced by external stimuli. The mitochondria respiratory chain is considered an important site of ROS production within mast cells [25]. An increased cellular level of ROS induced by CSE and PM2.5 is considered as an important factor for promoting lung diseases though regulation of the autophagy activity [16, 26, 27]. In colonic goblet cells, autophagy is necessary for orchestrating the cell functions by regulating the effect of NADPH oxidases (NOXs) on driving the ROS production [28, 29]. In the airway epithelium, autophagy promotes IL-13-mediated increase in superoxide levels by directing Dual Oxidase 1 (DUOX1) to the apical surface of the airway epithelium [30]. NOX4, a subtype of nonphagocytic NADPH oxidase, has a key role in the H_2_O_2_-induced MUC5AC hyperexpression in the airway epithelial cells [15]. In addition, DUOX1 also has a critical role in mucin expression in airway epithelial cells via PKCdelta/PKC-DUOX1-ROS-TACE-pro-ligand-EGF receptor cascade [31]. TGF-*β*1 triggers intracellular ROS release in ASMCs by upregulation of NOX4 and inhibition of MnSOD and catalase [32]. Furthermore, NOX4 upregulation in the cortex of diabetic rat kidney has been linked to glucose-induced mitochondrial ROS [33]. Collectively, these mechanisms propelled us to reveal the role of the ROS generation machinery in the airway mucus secretion in an autophagy-dependent fashion.

We hypothesized that ROS act as upstream of autophagy activation in response to TGF-*β*3 induction. Furthermore, ROS-induced autophagy is essential for airway mucus secretion dependent on NADPH oxidase in airway epithelial cells. In this paper, we discovered that persistent TGF-*β*3 activation increases ROS levels in a NOX4-dependent pathway and subsequently induces autophagy, as well as MUC5AC expression in the epithelial cells. The lack of NOX4 in the epithelial cells could reduce ROS generation and autophagy-dependent MUC5AC expression treated with TGF-*β*3.

## 2. Materials and Methods

### 2.1. Animals

C57BL/6J female mice, 6-8 weeks old, weighing 20-25 g, were housed in an environment with temperature of 22 ± 1°C, relative humidity of 50 ± 1%, and a light/dark cycle of 12/12 hrs. All animal studies (including the mice euthanasia procedure) were done in compliance with the regulations and guidelines of Southwest Medical University institutional animal care and conducted according to the AAALAC and the IACUC guidelines.

All animals were treated with 20 *μ*g HDM (Greer Laboratories, 326779) and 1 mg Al(OH)_3_ using i.p. injection. The same injections were repeated 7 days later. One week after the final injection, the mice were treated with HDM (10 *μ*g, i.n.) or combined with N-Acetyl-L-cysteine (NAC, Sigma-Aldrich, A9165; 3 mmol/kg, i.g.) for 7 days. At days 7 postchallenge, the mice were sacrificed, and their bronchoalveolar lavage fluid (BALF) and lung tissues were collected.

For blocking TGF-*β*3 signaling, TGF-*β*3-neutralizing antibodies (R&D Systems, AF-243-NA) and normal goat isotype control IgGs (R&D Systems, AB-108-C) were dissolved in PBS. Mice were given TGF-*β*3-neutralizing antibodies (30 *μ*g per mouse) or isotype control IgGs (30 *μ*g per mouse) i.p. daily 30 min before each challenge for 7 days. Mice were sacrificed 24 hours following the last challenge.

### 2.2. Cell Culture, Transfection, and Lentiviral Transduction

Human bronchial epithelial cells (16HBE cells) were cultured in high-glucose DMEM (HyClone, SH30022.01) supplemented with 10% fetal bovine serum (Corning, 35-076-CV), 50 U/ml penicillin, and 50 U/ml streptomycin in a humidified atmosphere containing 5% CO_2_ at 37°C. 16HBE cells were seeded in six-well plates and transfected using lipofectamine 2000 (Invitrogen, 11668019) and Optimem media (Gibco, 31985062) according to the manufacturer's protocol. The control siRNA and NOX4-siRNA were purchased from Santa Cruz Biotechnology (sc-41586).

Smad2-siRNA lentivirus vectors and Smad3-siRNA lentivirus vectors were purchased from GeneChem Technology (Shanghai, China). 16HBE cells (2 × 10^5^/ml) were transduced as previously described [24].

### 2.3. Drug Treatments

Cells were seeded at 0.5 million cells per well in six-well plates. Twenty-four hours after seeding, cells were treated with 10 mM NAC to prevent ROS formation and then (2 hours later) with TGF-*β*3 (PeproTech, 100-36E; 10 ng/ml) and incubated for an additional 24 hrs. Control cells were incubated with an equal amount of PBS.

### 2.4. RNA Isolation and Quantitative Real-Time PCR Analysis

Total cellular RNA was extracted using the RNAsimple Total RNA Kit (TIANGEN, DP419) and was reverse transcribed using the PrimeScript™ RT Master Mix (Takara, RR036A). RT-PCR was performed on a LightCycler 480 instrument, using SYBR Advantage qPCR Premix (Clontech, 639676). The primers used in the present study are listed in Table 1.

### 2.5. ELISA

ELISA assessed the levels of TGF-*β*3 in BALF in the mice using a reagent kit (Elabscience Biotechnology, E-EL-M1192) following the manufacturer's instructions.

### 2.6. Immunohistochemistry

Lung tissues were fixed in 10% neutral-buffered formalin for 24 hrs and then embedded in paraffin. Tissues were then cut in 5 *μ*m sections and stained with standard hematoxylin-eosin (H&E) staining. IHC assay was performed using antibodies to TGF-*β*3 (1 : 200; Santa Cruz, SC-82) according to a previously described approach [24].

### 2.7. Immunofluorescence Staining

For immunofluorescence (IF) analysis, the sections of lung tissues were fixed with 4% formaldehyde and permeabilized with 0.3% Triton X-100 in PBS for 10 min at room temperature. After blocking with 1% BSA, the specimens were incubated with primary antibodies to MUC5AC (1 : 100; Abcam, ab24070) at 4°C overnight. Subsequently, Alexa Fluor 488 (1 : 500; Invitrogen, A28175)- or Alexa Fluor 555 (1 : 500; Invitrogen, A32732)-conjugated secondary antibody was used to probe the primary Ab. DAPI was used for nuclear staining. Samples were then imaged using the SP5 Leica confocal microscope with Leica Application Suite Software (Version number 14.0.0.162, Leica, Germany).

### 2.8. Confocal Microscope

16HBE cells were plated in 6-well plates. After reaching 30%-50% confluence, cells were incubated in growth medium containing mCherry-EGFP-LC3 lentivirus (GeneChem Technology, Shanghai, China) at 37°C for 12 hrs, after which the media was replaced with the fresh one. The infected cells were selected with 1 *μ*g/ml puromycin for generation of stable cDNA-expressing cell lines.

To explore the role of ROS in TGF-*β*3-induced autophagy, the cells were treated with NAC (10 mM) for two hours and were then treated with TGF-*β*3 (10 ng/ml) for additional 24 hrs. To explore the role of NOX4 in TGF-*β*3-induced autophagy, the cells were grown in medium containing TGF-*β*3 and NOX4-siRNA at the indicated concentrations for 24 hrs at 37°C. LC3 puncta were examined with the SP5 Leica confocal microscope (Leica, Germany).

### 2.9. Western Blotting

The cells were lysed using RIPA buffer (Thermo Fisher Scientific, 87787) supplemented with protease inhibitor cocktail and phosphatase inhibitor cocktail (Thermo Fisher Scientific, 78420). Briefly, samples were left on ice for 30 min and then centrifuged at 12,000 g for 15 min at 4°C. Samples were then mixed with 5x SDS PAGE sample buffer (Beyotime, P0015L), heated to 95°C for 5 min, and separated on 12% SDS-PAGE gels. Consequently, the proteins were transferred to polyvinylidene difluoride membranes (Merck Millipore, ISEQ00010) and blocked with 5% nonfat dry milk, 0.05% Tween 20 in Tris-buffered saline for two hours. The blocked membranes were then incubated with primary antibody at 4°C overnight. Membranes were then washed three times with 0.05% Tween 20 in Tris-buffered saline and incubated with secondary antibody at 4°C for additional two hours. The membrane was imaged using the ChemiDoc Touch with Clarity Western ECL Substrate (Bio-Rad, 170-5061). The following primary antibodies were used for detection of target proteins at the indicated dilutions: anti-NOX4 mouse monoclonal antibody (Abcam, ab133303; 1 : 1000), anti-DUOX1 antibody (Santa Cruz, sc-393096; 1 : 1000), anti-LC3 antibody (Abcam, ab48394; 1 : 1000), anti-Smad2 antibody (CST, 5339S; 1 : 1000), anti-Smad3 antibody (Abcam, ab28379; 1 : 1000), anti-phospho-Smad2 antibody (CST, 3108S; 1 : 1000), anti-phospho-Smad3 antibody (Abcam, ab52903; 1 : 1000), and anti-GAPDH antibody (Beyotime, AF0006; 1 : 1000).

### 2.10. ROS Measurement

The ROS generation in 16HBE cells was measured using BBoxiProbeTM A Hydrogen Peroxide Assay Kit (BestBio, BB-47032) according to the manufacturer's instructions. In brief, the cells were exposed to PBS or TGF-*β*3 for 24 hrs. NAC, 10 mM, was used as a ROS inhibitor for each condition. After treatment, cells were washed with PBS and then incubated with 10 *μ*M BBoxiProbeTM A in DMEM for 30 min at 37°C. Cells were then washed with PBS, and the images were taken with a fluorescence microscope.

Frozen lung tissues were incubated with 25 *μ*M DCFH-DA (Beyotime, S0033) in PBS for 15 min at 37°C. The sections were washed with PBS for 3 min and then imaged using a fluorescence microscope.

### 2.11. Statistical Analysis

Statistical analyses were carried out using SPSS 16.0 software. All data are presented as mean ± s.d. Statistically significant differences were determined by one-way ANOVA test, Student's *t*-test, and nonparametric Mann-Whitney test. *P* values < 0.05 were considered statistically significant.

## 3. Results

### 3.1. Increasing Levels of ROS and TGF-*β*3 in HDM-Challenged Mice

**Figure 1:**
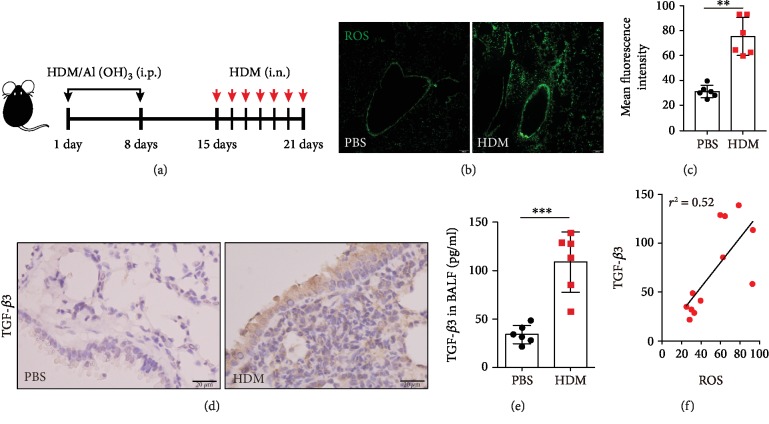
Increasing levels of ROS and TGF-*β*3 in HDM-challenged mice. (a) Schematic diagram of the experimental protocol for sensitization and challenge with HDM (*n* = 6 mice for each group). (b) Representative confocal laser immunofluorescence photomicrography of the lung tissues in the PBS-challenged and HDM-challenged mice showed the ROS generation in the airway epithelial cells. (c) The fold change of ROS signal intensity is shown. (d) Representative images of H&E-stained lung tissue sections of PBS-challenged and HDM-challenged mice showing TGF-*β*3 expressions. (e) TGF-*β*3 in BALF was detected by ELISA. (f) The relevance between TGF-*β*3 and ROS generation was tested with the Pearson correlation test (^∗∗^*P* < 0.01). Each point is an individual mouse. Data are presented as means ± s.d.^∗^*P* < 0.05, ^∗∗^*P* < 0.01, and ^∗∗∗^*P* < 0.001, determined by an unpaired *t*-test.

Previous studies have suggested a direct relationship between Th2 inflammation, autophagy, and ROS [18]. To evaluate the relationship between TGF-*β*3 and ROS levels, we used an allergen-driven airway disease model that was challenged with intratracheal instillation HDM after intraperitoneal sensitization (Figure 1(a)). Briefly, twenty-four hours after exposure to HDM, DCFH-DA probe was used to detect ROS levels, and increased ROS levels were observed in the airways (Figures 1(b) and 1(c)).

Previous studies have examined the transcriptional response of human lens epithelial cells to TGF-*β* and uncovered a potential source of the TGF-*β*-driven ROS production and subsequent oxidative stress [21]. In this study, we examined an increase in TGF-*β*3 expression in the lung tissues of the mice exposed to HDM (Figure 1(d)), learning the BALF from these mice actually contained greater TGF-*β*3 production (Figure 1(e)). As expected, we found a great link between TGF-*β*3 and ROS levels. Based on these data, we concluded that the TGF-*β*3 expression was associated with increased ROS production in airway epithelial cells in allergic-mice models.

### 3.2. TGF-*β*3 Increases ROS Levels in Epithelial Cells via NOX4

**Figure 2:**
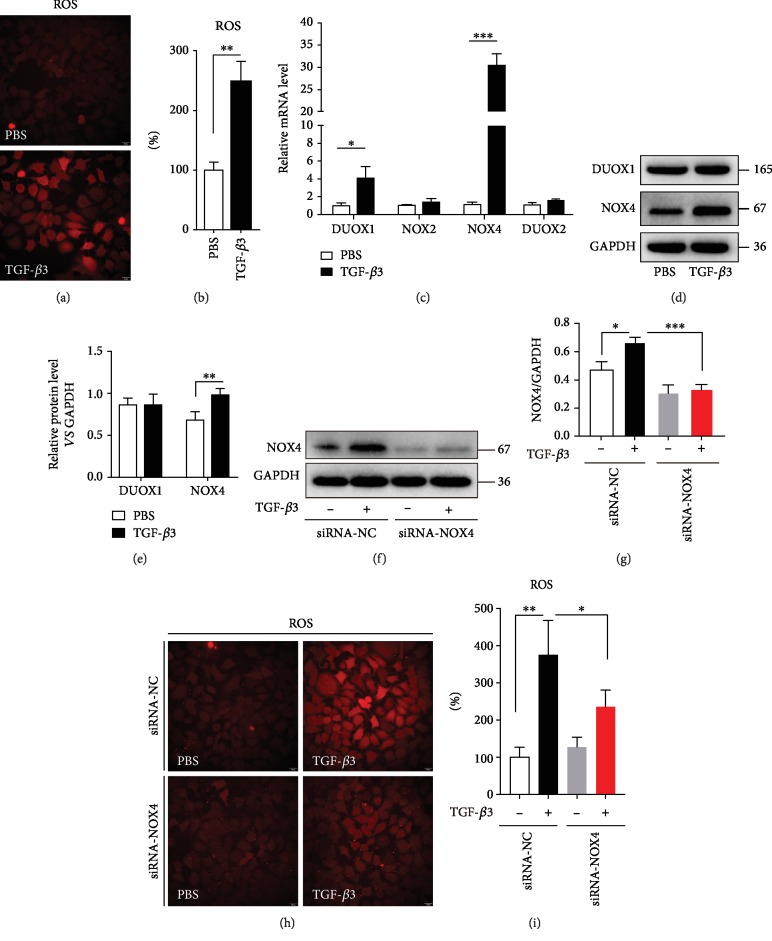
TGF-*β*3 increases ROS levels in airway epithelial cells via NOX4. (a, b) 16HBE cells were treated with TGF-*β*3 (10 ng/ml) for 24 hrs, and then, the ROS generation was measured using the oxidant sensitive fluorometric probe BBoxiProbeTM A. Representative images of BBoxiProbeTM A probe fluorescent signal and the fold change of ROS signal intensity are shown. (c) Real-time PCR was performed to detect the expression of DUOX1, DUOX2, NOX2, and NOX4 after treatment with TGF-*β*3 (10 ng/ml). (d, e) Then, the expression of DUOX1 and NOX4 was detected using western blot assays. And relative changes in the density of DUOX1 and NOX4 were detected. (f) 16HBE cells were transfected with NOX4-siRNA. After treating the cells with TGF-*β*3 (10 ng/ml) for 24 hrs, NOX4 was detected by western blot. (g) Relative changes in the density of NOX4. (h, i) 16HBE cells were transfected with NOX4-siRNA. After treating the cells with TGF-*β*3 (10 ng/ml) for 24 hrs, the ROS generation was measured using the oxidant-sensitive fluorometric probe BBoxiProbeTM A. Representative images of BBoxiProbeTM A probe fluorescent signal and the fold change of ROS signal intensity are shown. Data are representative of the three independent experiments and are presented as means ± s.d.^∗^*P* < 0.05, ^∗∗^*P* < 0.01, and ^∗∗∗^*P* < 0.001, determined by Student's *t*-test or one-way ANOVA with Tukey-Kramer posttest.

Systemic and airway-associated oxidative stress is increased in asthmatic patients compared with healthy individuals [34]. ROS production has been recognized to have a central role in a range of asthmatic pathophysiological processes [35]. Our data found that TGF-*β*3 could increase ROS levels in the human bronchial epithelial cells (16HBE cells) (Figures 2(a) and 2(b)).

DUOX1 is upregulated in epithelial cells in asthmatics [36] and has been identified as a major ROS driver in the airway epithelial cells [31, 37, 38]. Unexpectedly, although TGF-*β*3 could induce DUOX1 mRNA levels in the epithelial cells, it did not affect the total DUOX1 protein levels (Figures 2(c)–2(e)). Moreover, DUOX2 mRNA levels were not affected by the TGF-*β*3 stimulation in the epithelial cells (Figure 2(c)). It has been suggested that NOX4-generated ROS underlie AHR by mediating airway smooth muscle hypercontractility [39]. In our present study, we found that TGF-*β*3 not only induced NOX4 mRNA levels but also upregulated the total NOX4 protein levels (Figures 2(c)–2(e)). However, NOX2 mRNA levels, another NADPH oxidase protein, were not affected by the TGF-*β*3 stimulation in the epithelial cells (Figure 2(c)). To measure the effect of NOX4 on the oxidant levels in the epithelial cells, we used NOX4 small interferon RNA to find that the inhibition of NOX4 could significantly decrease the ROS levels in the epithelial cells treated by TGF-*β*3 (Figures 2(f)–2(i)). These studies indicated that NOX4 was involved in TGF-*β*3-induced ROS production in the epithelial cells.

### 3.3. TGF-*β*3-Induced ROS Production Triggers Autophagy and Upregulates MUC5AC in the Airway Epithelial Cells

**Figure 3:**
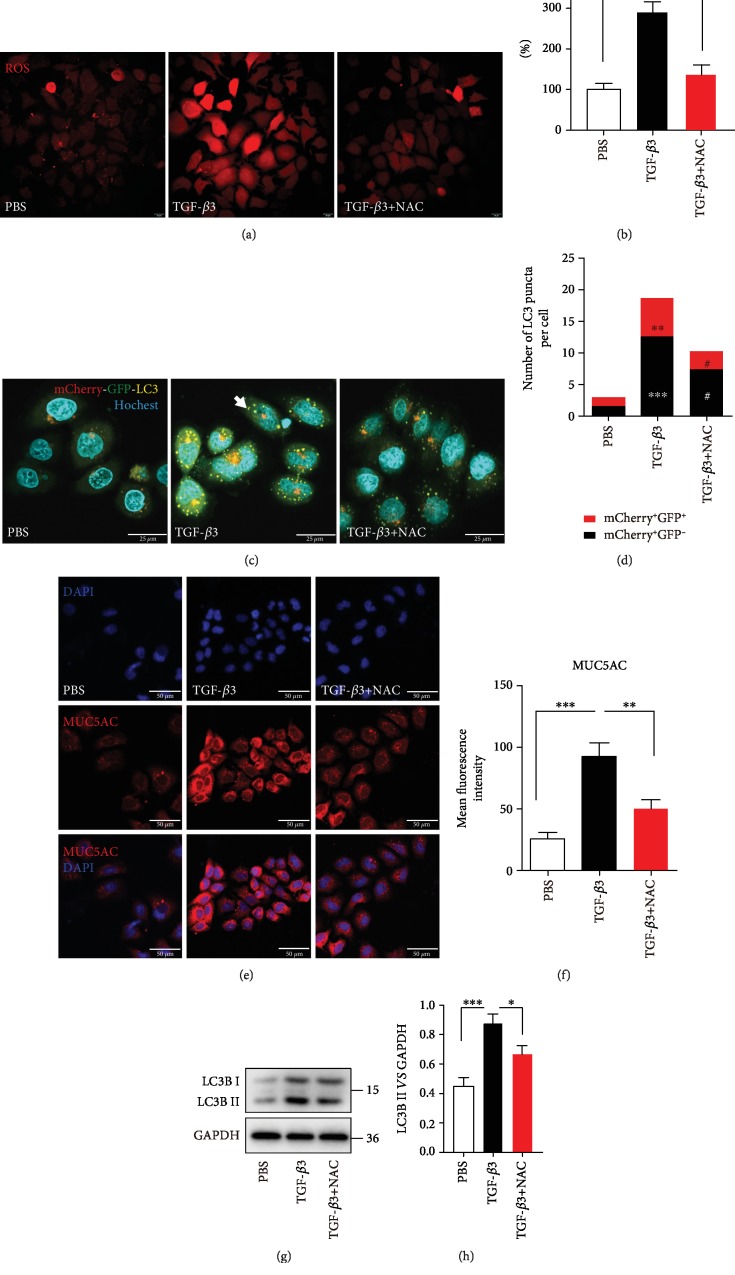
TGF-*β*3-induced ROS production triggers autophagy and upregulates MUC5AC in the airway epithelial cells. (a, b) 16HBE cells were incubated with NAC for 2 hrs and then treated with TGF-*β*3 for another 24 hrs. And then, the ROS generation was measured using the oxidant-sensitive fluorometric probe BBoxiProbeTM A. Representative images of BBoxiProbeTM A probe fluorescent signal and the fold change of ROS signal intensity are shown. (c) 16HBE cells that stably expressed mCherry-EGFP-LC3 fusion protein were incubated with NAC for 2 hrs and then treated with TGF-*β*3 for another 24 hrs. In green- and red-merged images, autophagosomes are shown as yellow puncta (i.e., mCherry^+^EGFP^+^), while autolysosomes are shown as red puncta (i.e., mCherry^+^EGFP^−^). Autophagic flux was increased when both yellow and red puncta are increased in the cells. Confocal microscopic analysis was shown (×1000 magnification). (d) Quantification of the number of LC3 puncta (each group *n* = 10 images for quantification). (e) 16HBE cells were incubated with NAC for 2 hrs and then treated with TGF-*β*3 for another 24 hrs. Representative immunofluorescence images of TGF-*β*3-induced MUC5AC in 16HBE cells treated with NAC. (f) Quantitation of the fluorescence intensity of MUC5AC. (g, h) LC3B was detected by western blot. And relative changes in the density of LC3B II were detected. Data are representative of three independent experiments and are presented as means ± s.d.^∗^*P* < 0.05, ^∗∗^*P* < 0.01, and ^∗∗∗^*P* < 0.001, TGF-*β*3 *vs*. PBS; ^#^*P* < 0.05 and ^##^*P* < 0.01, TGF-*β*3+NAC *vs*. TGF-*β*3, determined by one-way ANOVA with Tukey-Kramer posttest.

Oxidant stress is a well-recognized activator of autophagy [9, 40]. Our data suggested that the inhibition of ROS production by NAC in TGF-*β*3-activated epithelial cells significantly decreased autophagy flux (Figures 3(a)–3(d)). We previously found that TGF-*β*3 stimulation in the epithelial cells was associated with an increase in mucus production and autophagy was required for TGF-*β*3-induced MUC5AC levels in 16HBE cells [24]. In this study, immunofluorescence staining revealed that MUC5AC protein was significantly reduced in 16HBE cells treated with TGF-*β*3 and NAC compared to 16HBE cells treated with TGF-*β*3 only (Figures 3(e) and 3(f)). Then, the western blotting results showed that inhibition of ROS generation reduced LC3B-II protein expression in the epithelial cells with TGF-*β*3 treatment condition (Figures 3(g) and 3(h)). These findings imply that oxidant stress is involved in the TGF-*β*3-induced mucus production and autophagy activity.

**Figure 4:**
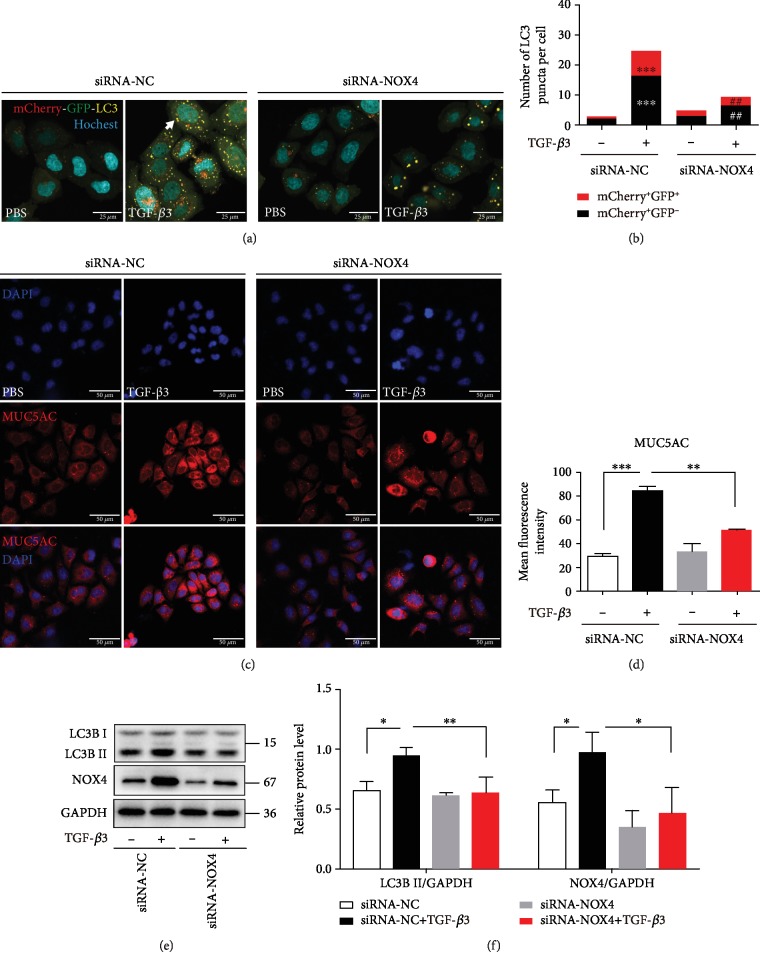
NOX4 is required for autophagy activity and MUC5AC expression in TGF-*β*3 stimulation. (a) 16HBE cells that stably expressed mCherry-EGFP-LC3 fusion protein were transfected with NOX4-siRNA. After treating with TGF-*β*3 (10 ng/ml) for 24 hrs, autophagosomes were observed under a confocal microscope (×1000 magnification). (b) Quantification of the number of LC3 puncta (each group *n* = 10 images for quantification). (c, d) 16HBE cells were transfected with NOX4-siRNA. After treating the cells with TGF-*β*3 (10 ng/ml) for 24 hrs, MUC5AC were detected by immunofluorescence. (e, f) LC3B and NOX4 were detected by western blot. And relative changes in the density of LC3B II and NOX4 were detected. Data are representative of three independent experiments and are presented as means ± s.d.^∗^*P* < 0.05, ^∗∗^*P* < 0.01, and ^∗∗∗^*P* < 0.001, TGF-*β*3+siRNA-NC *vs*. PBS+siRNA-NC; ^#^*P* < 0.05 and ^##^*P* < 0.01, TGF-*β*3+siRNA-NOX4 *vs*. TGF-*β*3+siRNA-NC, determined by one-way ANOVA with Tukey-Kramer posttest.

In order to further examine the role of oxidant stress in the production of TGF-*β*3-induced MUC5AC expression in airway epithelial cells, small interferon RNA of NOX4 was applied to reduce the ROS production. Knockdown of NOX4 reduced the autophagy flux in the epithelial cells treated with TGF-*β*3 (Figures 4(a) and 4(b)). Furthermore, immunofluorescence staining revealed that the lack of NOX4 markedly inhibited the MUC5AC expression induced by TGF-*β*3 in the epithelial cells (Figures 4(c) and 4(d)). As anticipated, the lack of NOX4 markedly inhibited the LC3B-II levels induced by TGF-*β*3 in the epithelial cells (Figures 4(e) and 4(f)). Inhibition of ROS generation levels could reduce autophagy activation and MUC5AC expression treated with TGF-*β*3 in airway epithelial cells (Figures 3(g) and 3(h)). Collectively, our present observations demonstrated that NOX4 is required for autophagy activity and MUC5AC expression in TGF-*β*3 stimulation.

### 3.4. TGF-*β*3 Enhanced the Expression of NOX4 by Smad2/3 Pathway

**Figure 5:**
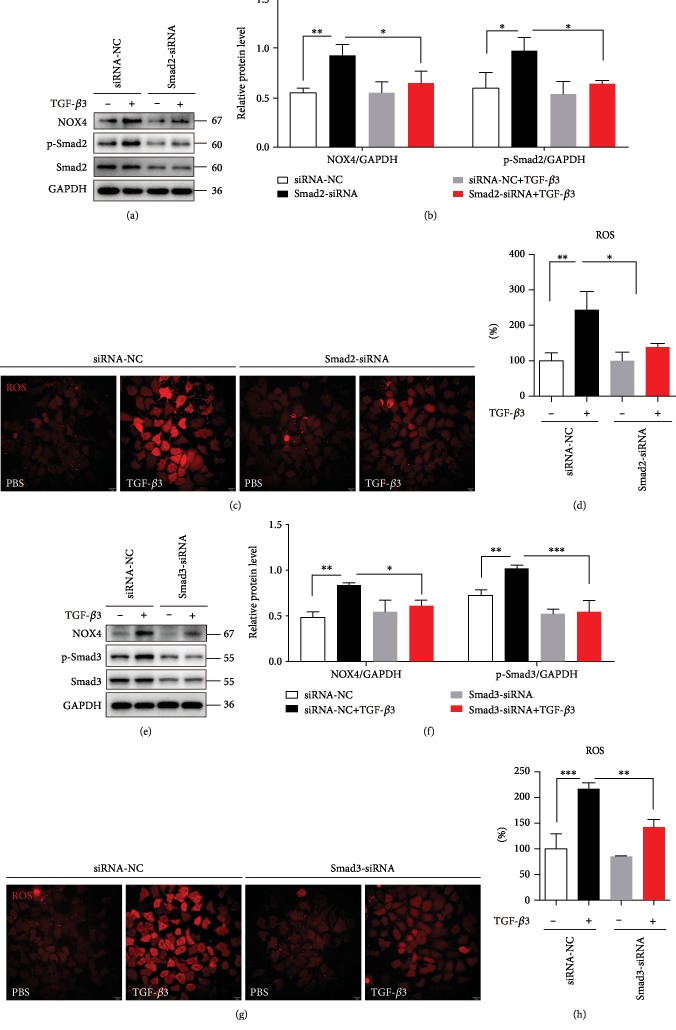
TGF-*β*3 enhanced the expression of NOX4 by Smad2/3 pathway. (a, b) 16HBE cells were transfected with Smad2-siRNA lentivirus. After treating the cells with TGF-*β*3 (10 ng/ml) for 24 hrs, NOX4, Smad2, and phospho-Smad2 were detected by western blot. And relative changes in the density of NOX4 and phospho-Smad2 were detected. (c, d) The ROS generation was measured using the oxidant sensitive fluorometric probe BBoxiProbeTM A. Representative images of BBoxiProbeTM A probe fluorescent signal and the fold change of ROS signal intensity are shown. (e, f) 16HBE cells were transfected with Smad3-siRNA lentivirus. After treating the cells with TGF-*β*3 (10 ng/ml) for 24 hrs, NOX4, Smad3, and phospho-Smad3 were detected by western blot. And relative changes in the density of NOX4 and phospho-Smad3 were detected. (g, h) The ROS generation was measured using the oxidant-sensitive fluorometric probe BBoxiProbeTM A. Representative images of BBoxiProbeTM A probe fluorescent signal and the fold change of ROS signal intensity are shown. Data are representative of the three independent experiments and are presented as means ± s.d.^∗^*P* < 0.05, ^∗∗^*P* < 0.01, and ^∗∗∗^*P* < 0.001, determined by one-way ANOVA with Tukey-Kramer posttest.

Previous study had demonstrated that Smad2/3 is involved in TGF-*β* inducing ROS generations [41]. In our present study, we found that NOX4 was required for ROS generation induced by TGF-*β*3. Thus, the association between Smad2/3 and NOX4 needs to be assessed. Cells were treated with siRNA against Smad2 or Smad3. The results indicated that Smad2-siRNA and Smad3-siRNA significantly decreased NOX4 expression induced by TGF-*β*3 (Figures 5(a)–5(h)). Meanwhile, effects of Smad2 and Smad3 knockdown on the expression of ROS were evaluated. We found that Smad2 and Smad3 knockdown significantly decreased ROS generation (Figures 5(a)–5(h)). These results suggested that Smad2/3 signaling serves a key function in the regulation of NOX4 expression and ROS generation induced by TGF-*β*3.

### 3.5. NAC Inhibits Autophagy Activation and MUC5AC Expression in Asthma Mice Models

**Figure 6:**
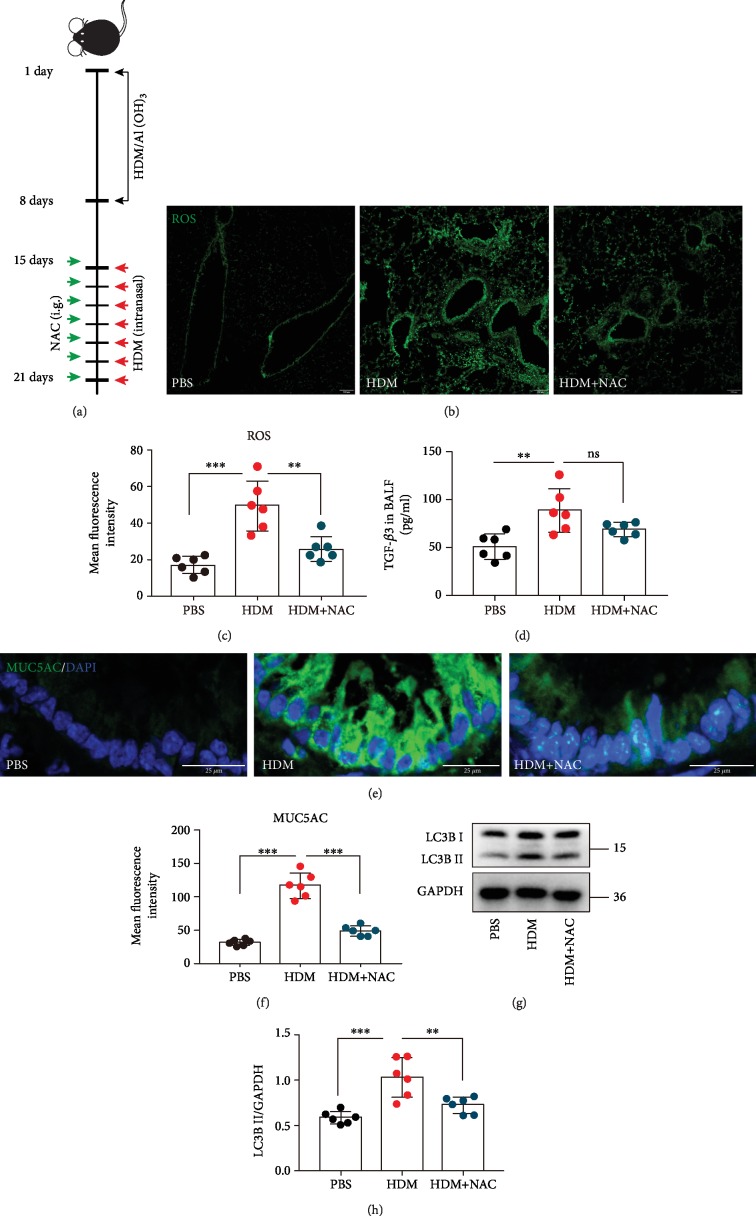
NAC inhibits autophagy activation and MUC5AC expression in asthma mice models. (a) Schematic diagram of the experimental protocol for sensitization and challenge with HDM and the experimental protocol for the mice pretreated with NAC (*n* = 6 mice for each group). (b) Representative confocal laser immunofluorescence photomicrography of the lung tissues in the mice showed the ROS generation in the lung tissues. (c) The fold change of ROS signal intensity is shown. (d) TGF-*β*3 in BALF was detected by ELISA. (e) Representative immunofluorescence images of MUC5AC expression in the airway epithelial cells of the mice. (f) Quantitation of the fluorescence intensity of MUC5AC. (g) The expression of LC3B was detected using western blot assays. (h) Relative changes in the density of LC3B II. Each point is an individual mouse. Data are presented as means ± s.d.^∗^*P* < 0.05, ^∗∗^*P* < 0.01, and ^∗∗∗^*P* < 0.001, determined by one-way ANOVA with Tukey-Kramer posttest.

To further explore the effect of excessive ROS generation on autophagy activation and the downstream MUC5AC expression, we used NAC to inhibit the oxidant stress in asthma mice models (Figure 6(a)). We collected lung tissues dissociated from the mice to directly measure oxidant levels and TGF-*β*3 levels. In the lung tissues of the HDM-challenged mice, there was a significant increase in ROS generation detected by immunofluorescence staining (Figures 6(b) and 6(c)). A decrease of ROS generation was specifically identified in the lung tissues of mice with NAC before treatment with HDM (Figures 6(b) and 6(c)). Furthermore, there was a significant increase in TGF-*β*3 generation (Figure 6(d)). However, NAC could not suppress the levels of TGF-*β*3 (Figure 6(d)). As expected, an elevation in ROS levels was accompanied by increased MUC5AC expression (Figures 6(e) and 6(f)) and autophagy activity (Figures 6(g) and 6(h)) after treatment with HDM, while an inhibition of ROS generation by NAC revealed a decrease in MUC5AC expression (Figures 6(e) and 6(f)) and autophagy activity (Figures 6(g) and 6(h)). These consequences suggested that oxidant stress has a vital role in triggering autophagic activity and subsequently MUC5AC expression in the asthma mice models.

### 3.6. Neutralization of TGF-*β*3 Significantly Inhibited ROS Production, Autophagy Activation, and MUC5AC Expression in Asthma Mice Models

**Figure 7:**
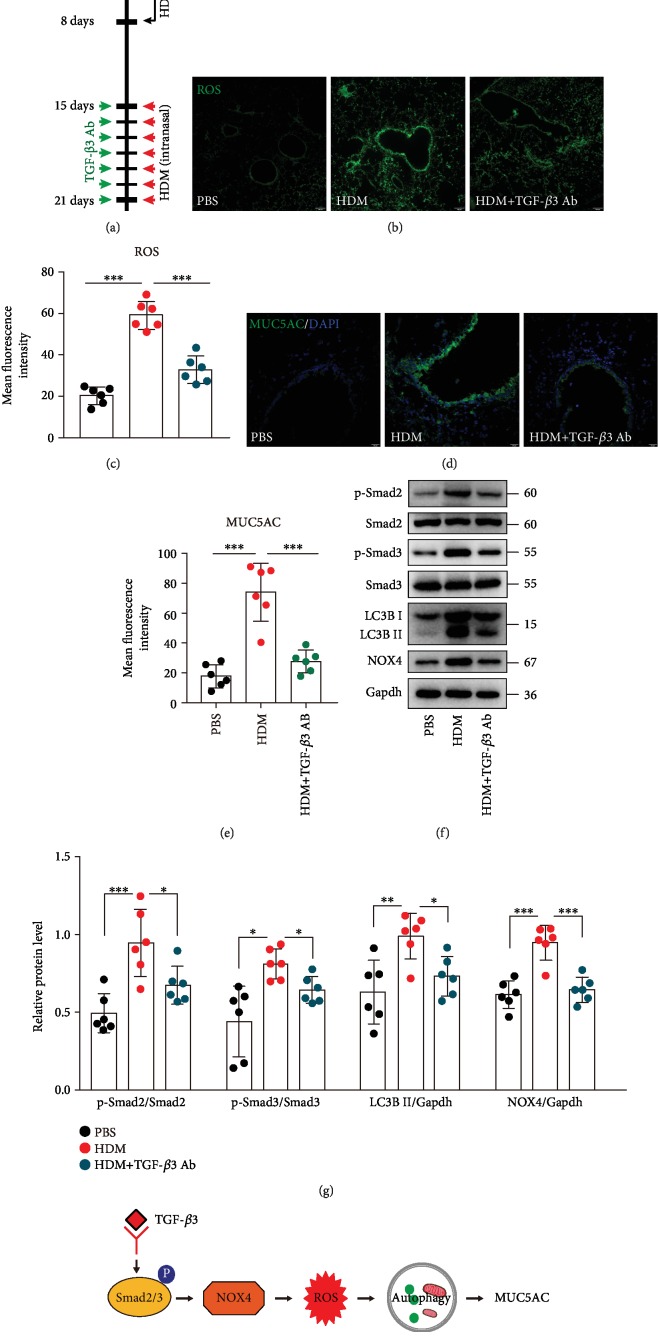
Neutralization of TGF-*β*3 significantly inhibited ROS production, autophagy activation, and MUC5AC expression in asthma mice models. (a) Schematic diagram of the experimental protocol for sensitization and challenge with HDM and the experimental protocol for the mice pretreated with TGF-*β*3-neutralizing antibody or isotype control IgGs (*n* = 6 mice for each group). (b) Representative confocal laser immunofluorescence photomicrography of the lung tissues in the mice showed the ROS generation in the lung tissues. (c) The fold change of ROS signal intensity is shown. (d) Representative immunofluorescence images of MUC5AC expression in the airway epithelial cells of the mice. (e) Quantitation of the fluorescence intensity of MUC5AC. (f) The expression of phospho-Smad2, Smad2, phospho-Smad3, Smad3, LC3B, and NOX4 was detected using western blot assays. (g) Relative changes in the density of phospho-Smad2, phospho-Smad3, LC3B II, and NOX4. Each point is an individual mouse. Data are presented as means ± s.d.^∗^*P* < 0.05, ^∗∗^*P* < 0.01, and ^∗∗∗^*P* < 0.001, determined by one-way ANOVA with Tukey-Kramer posttest. (h) Schematic diagram of the mechanisms of the requirement of the NOX4-mediated ROS for autophagosome and NOX4 has been identified as a major driver in the epithelial cells by TGF-*β*3 treatment.

To determine the contribution of TGF-*β*3 in induction of ROS and autophagy and MUC5AC expression in asthma mice models, we examined the effects of a TGF-*β*3-neutralizing antibody on ROS generation, autophagy activation, and MUC5AC expression of mice after the seventh HDM challenge. HDM-challenged mice were administered i.p. either the TGF-*β*3-neutralizing antibody (30 *μ*g per mouse, i.p.) or an isotype control IgG (goat IgG, 30 *μ*g per mouse, i.p.) daily, starting from day 15 until day 21 (Figure 7(a)). Unchallenged mice received isotype control IgGs (i.p.) only. TGF-*β*3-neutralizing antibody significantly reduced the ROS generation in the airways of OVA-treated mice compared to isotype IgG antibodies (Figures 7(b) and 7(c)). Immunofluorescence staining revealed reduced MUC5AC expression in anti-TGF-*β*3 Ab-treated asthma mice models (Figures 7(d) and 7(e)). As anticipated, western blotting results showed that the levels of LC3B-II were markedly reduced in the lung tissues of the anti-TGF-*β*3 Ab-treated mice (Figures 7(f) and 7(g)). In addition, TGF-*β*3-neutralizing antibody also decreased the expression of NOX4, as well as the phosphorylation of Smad2 and Smad3 (Figures 7(f) and 7(g)). Taken together, these data suggest that TGF-*β*3 signaling is involved in ROS production, autophagy activation, and MUC5AC expression in the asthma mice models. Collectively, our data suggested that TGF-*β*3 induces autophagy activity and downstream MUC5AC expression in the epithelial cells (Figure 7(h)). We have now extended this observation to identify the requirements of the NOX4-mediated ROS for autophagosome (Figure 7(h)). NOX4 was identified as a major driver in the epithelial cells following TGF-*β*3 treatment (Figure 7(h)).

## 4. Discussion

In this study, we showed that TGF-*β*3 stimulates ROS and that this signaling has physiological consequences. TGF-*β*3 exposure initially triggers the production of ROS and subsequently elicits autophagy in airway epithelial cells, which then regulate MUC5AC expression. Additionally, we found that TGF-*β*3 regulates ROS expression dependent on NOX4, rather than DUOX1 or NOX2. These results suggest that TGF-*β*3-induced ROS generation is essential for the development and maintenance of the asthmatic airway environment.

ROS have emerged as key signaling intermediates in many vital cellular processes including mitogenesis, gene expression, stress responses, and ion channel function as well as adhesion and motility [25, 42]. Several reports have established a link between cytokines and ROS formation in the lung diseases [19, 30, 43, 44]. These studies have shown that ROS are important for the pathogenesis of inflammation and tissue injury [45]. IL-13 is increased in asthma and other airway diseases contributing to chronic inflammation, which increases reactive oxygen species (ROS) in airway epithelial cells in an autophagy-dependent fashion [30]. Furthermore, IL-13 also functions as a driver of mucus hypersecretion in the airway epithelia in autophagy-related asthma [18]. TGF-*β* is an inflammatory mediator that is produced at higher basal levels in asthmatic airways and is several-folds increased after allergens reach levels that correlate with the severity of AHR and remodeling [46–48]. Previous studies have demonstrated that TGF-*β* contributes to oxidative stress by increasing ROS production through NOX induction [49]. Evidence has shown that ROS can influence TGF-*β* signaling via various pathways, including the Smad pathway, MAPK (such as p38) pathway, and Rho-GTPase pathway [50]. Our previous study has demonstrated that TGF-*β*3 induces autophagy activity and that downstream mucus secretion is Smad2/3 signaling pathway-dependent [24]. Collectively, these studies urged us to further investigate TGF-*β*3-induced mucus secretion in asthma via ROS-dependent mechanism. In our study, we found that increased TGF-*β*3 in the lung tissue in the HDM-challenged mice was correlated with the excessive ROS generation. These results implied the existence of a relationship between TGF-*β*3-mediated downstream mechanisms and ROS generation in the epithelial cells.

ROS are important second messengers involved in the cellular signal-transduction pathways [51]. Multiple enzyme systems cause the ROS generation, among which the NOXs are the main producers of ROS [52, 53]. NOX enzymes are a family of NADPH-dependent oxygen reductases that are widely expressed in eukaryotes [54]. TGF-*β* is widely expressed in inflammatory cells infiltrated in the bronchial mucosal but also in structural cells of the airway wall including epithelial and endothelial cells [24]. TGF-*β* induces the epithelial-to-mesenchymal transition (EMT) associated with NOX4, which is a TGF-*β*/Smad3-inducible source of reactive oxygen species (ROS) affecting cell migration and fibronectin expression [55]. Furthermore, NOX4 expression is increased in airway smooth muscle in asthma, leading to increased ROS production and intrinsic airway smooth muscle hypercontractility [39]. In asthma, the expression of DUOX1 increased, and the underlying mechanism of IL-13-mediated increase in superoxide levels is that autophagy pathway could direct DUOX1 to the apical surface of the airway epithelium [30]. In addition, TGF-*β*3 induced autophagy activity involved in Smad2/3 pathway [24]. In this study, we also found that TGF-*β*3 could induce NOX4 expression and ROS generation. Moreover, TGF-*β*3 could induce DUOX1 mRNA levels in the 16HBE cells. However, TGF-*β*3 did not affect the total DUOX1 protein levels. Genetic blockade of NOX4 exhibited a decrease of ROS generation induced by TGF-*β*3. Moreover, Smad2/3 is required for NOX4 expression and ROS generation induced by TGF-*β*3. These consequences demonstrated that Smad2/3 could be associated with TGF-*β*3 induced ROS generation by promoting NOX4.

Evidence has shown that NOX4-derived ROS are implicated in TGF-*β*-mediated cell migration, proliferation, hypertrophy, wound healing, and modulation of EMT markers in a variety of cell types [56–62]. Interestingly, we recently found that TGF-*β*3-induced autophagy contributed to increased MUC5AC production by activating activator protein-1 (AP-1) pathway [24]. Given that autophagy pathway is activated by oxidant stress [9, 40], in the present study, we further identified NOX4 as a major source of TGF-*β*3-induced ROS production that functions as an important factor in autophagy activity and downstream MUC5AC hyperexpression in the epithelial cells in asthma mice models.

In conclusion, we emphasize that TGF-*β*3 also has a critical role in regulating NOX4-dependent ROS-mediated cell signaling. Our observations suggest that ROS is an important aspect of biological effect of TGF-*β*3 in modulating autophagy activity. Moreover, in this study, we found evidence supporting the contribution of ameliorating autophagy by targeting inhibition of ROS generation.

## Figures and Tables

**Table 1:** The primers used for real-time qPCR.

Genes	Forward primer	Reverse primer
Human *DUOX1*	TTCACGCAGCTCTGTGTCAA	AGGGACAGATCATATCCTGGCT
Human *DUOX2*	CTGGGTCCATCGGGCAATC	GTCGGCGTAATTGGCTGGTA
Human *NOX2*	CAAGATGCGTGGAAACTACCTAAGAT	TCCCTGCTCCCACTAACATCA
Human *NOX4*	TCTGTTGTGGACCCAATTCA	AGCTGATTGATTCCGCTGAG
Human *GAPDH*	AGGTCGGAGTCAACGGATTTG	CATGGGTGGAATCATATTGGAACA

## Data Availability

The data used to support the findings of this study are available from the corresponding authors upon request.
